# Comparative Analysis of the Microbial Profiles in Supragingival Plaque Samples Obtained From Twins With Discordant Caries Phenotypes and Their Mothers

**DOI:** 10.3389/fcimb.2018.00361

**Published:** 2018-10-16

**Authors:** Yuqiao Zheng, Meng Zhang, Jin Li, Yuhong Li, Fei Teng, Han Jiang, Minquan Du

**Affiliations:** ^1^Hubei-Most KLOS&KLOBM, School & Hospital of Stomatology, Wuhan University, Wuhan City, China; ^2^Qingdao Institute of Bioenergy and Bioprocess Technology, Chinese Academy of Sciences, Shandong, ref-listChina

**Keywords:** early childhood caries, supragingival plaque, twins, oral microbiome, mother

## Abstract

Early childhood caries (ECC), the most frequent disease of oral cavity in preschool children, is the consequence of microbial, genetic, biochemical, socioeconomic, physical, environmental and health-influencing behavioral factors. To investigate the role of the oral microbiome and the impact of host and environmental factors in the occurrence and development of ECC, we studied the supragingival plaques of 14 twin pairs and a set of triplets with discordant caries phenotypes and 15 mothers, applying the Human Oral Microbe Identification using Next Generation Sequencing technique (HOMINGS). A total of 2,293,650 reads revealed 11 phyla, 116 genera, and 139 species of micromiome. Comparative analysis between the caries and caries-free group at species level revealed that the relative abundance of *Streptococcus mutans, Lactobacillus fermentum, Actinomyces islaelii, Neisseria sica*, and *Veilonella dispar* was much higher in caries group (*P* < 0.0001). Furthermore, monozygotic twins exhibited a higher degree of similarity than dizygotic twins. Finally, we analyzed the relationship between environmental factors and the oral microbiome, and our results indicat that the frequency of taking sweet food is associated with ECC. We conclude the following. First, the occurrence of *Streptococcus mutans, Lactobacillus fermentum, Neisseria sica*, and *Veilonella dispar* is strongly associated with the occurrence of ECC. Second, host genetic factors influence the oral microbiome composition, while environmental and behavioral factors like the frequency of taking sweet foods have an impact on the distribution of caries-related bacteria.

## Introduction

Dental caries represent one of the most common infectious diseases of the oral cavity among preschool children (Islam et al., 2007). Early childhood caries (ECC) is defined as “the presence of one or more decayed (non-cavitated or cavitated), missing teeth (due to caries), or filled tooth surfaces in any primary tooth in a child 71 months of age or younger” (Dentistry and Pediatrics, 2011). The incidence of dental caries among 5-years-old Chinese children has reached 66%, according to the Third National Epidemiological Survey on Oral Health conducted in 2005 (Ma et al., 2015), which suggests that ECC is still a significant public health problem in China. ECC not only impairs children's oral health, but it also has an impact on the future general growth and cognitive abilities of children (MartinsJúnior et al., 2013), so it is important to identify the risk factors for ECC and to develop effective preventive strategies. Increasing evidence suggests that the etiology of ECC involves a complex interplay of microbiological risk factors, feeding practices, sugars and socioeconomic factors (Colak et al., 2013). Recent studies have shown that the dental plaque microbiome closely correlates with severe ECC (Marsh, 2010), and it is well-established that certain components of microbiota, especially *Streptococcus mutans* and *Streptococcus sobrinus*, are key etiological factors in the initiation and progression of caries in oral cavity (Tanzer et al., 2001; Nurelhuda et al., 2010). More than 700 bacterial species reside in human oral cavity, many of which have not been cultured yet (Paster et al., 2001; Aas et al., 2005). Traditional methods that depend on bacterial cultures cannot identify the complete microbial profile of oral cavity. Recently developed molecular biology techniques, such as 16S rRNA gene pyrosequencing, have been applied in a wide range of human microbiome studies. A recent study employing 16S rRNA gene pyrosequencing technology revealed that patients who suffer from ulcerative colitis show dysbiosis in their gut bacterial communities (Walujkar et al., 2014). Moreover, the characteristics of the oral microbiome in patients with or without caries have been analyzed using 16S rRNA sequencing (Aas et al., 2008). The Human Oral Microbe Identification using Next Generation Sequencing (HOMINGS), the next generation of HOMIM (Aas et al., 2008), utilizes the speed and efficiency of Next Generation Sequencing (NGS) and it was developed by investigators at the Forsyth Institute (Cambridge, MA) in 2014, using specially designed probes to identify nearly 600 oral bacterial species. This approach, based on sequencing of the 16S rRNA gene, has been extensively validated (Belstrøm et al., 2016a,b; Daniel et al., 2016; Mougeot et al., 2016), and it has been demonstrated to be precise and useful in oral microbiome researches.

The oral microbiome plays a role in the pathogenesis of caries. Moreover, a number of aciduric dental plaque bacteria increase the formation of caries simultaneously (Vieira et al., 2014). However, besides oral bacteria, host genetic, behavioral and environmental determinants all play a role in determining the susceptibility to ECC (Ramos-Gomez et al., 2002). Twin studies have long been recognized for their value in uncovering the etiology of diseases and for their potential to distinguish genetic from environmental causes (Carlin et al., 2005). Therefore, various models have been extensively used to analyze twins in etiological studies. Investigators performed a comparative longitudinal study about fecal microbiomes of monozygotic and dizygotic twins who became discordant for kwashiorkor and their results suggested that the gut microbiome was a causal factor in kwashiorkor (Smith et al., 2013). In another research study (Lovelina et al., 2012), 9 monozygotic and 21 dizygotic twin pairs who were studied, and it was shown that genes play a role in the etiology of dental caries, periodontal disease and malocclusion. These findings have confirmed the importance of twin models to uncover genetic factors in etiological analysis. However, mothers constitute an essential component of the environmental factors linked to ECC. One study aimed to determine the moment of maternal transmission of *S. mutans*, and the results indicated that *S. mutans* is acquired by infants during a defined time window (Caufield et al., 1993). Priyadarshini (Priyadarshini et al., 2013) has shown that there is a strong association between maternal and child salivary *S. mutans* levels. However, most investigations of maternal bacterial transmission have focused only on *S. mutans*, and studies profiling the entire oral microbiome of mother child pairs are rare.

In this study, we have used HOMINGS to quantify the bacterial communities of 14 pairs of twins and a set of triplets with discordant caries phenotypes and of 15 mothers, exploring the role of oral microbiome and the impact of inheritance and environmental factors on the etiology of ECC.

## Materials and methods

### Ethics statement

Informed written consents were obtained from parents of all participating twins prior to enrollment, and all study procedures were approved by the Ethics Committee of the School & Hospital of Stomatology, Wuhan University (Wuhan, China).

### Subject enrollment

A total of 31 Chinese children aged 3–6 years (46–71 months), including 14 pairs of twins and a set of triplets (five twin pairs and a set of triplets were monozygotic, the remaining twins were dizygotic see Supplemental Table 1), as well as their mothers, grouped as mother-child pairs, were enrolled in our study. The demographic characteristics of the participants are shown in Table 1 below. Subjects came from various kindergartens in Wuhan, China. To be enrolled in our study, one member of the twin pair had to have two or more carious teeth, while the teeth of the other twin had to be completely healthy. All were children with primary dentition, and all had at least 18 teeth in the oral cavity. The decayed, missing and filled teeth (DMFT) and decayed, missing and filled tooth surfaces (DMFS) caries diagnostic criteria and scores were used to record the ECC status, in accordance with the World Health Organization guidelines (Dentistry and Pediatrics, 2011). Subjects in the caries group had two or more cavitated teeth and individuals in caries-free group did not have any carious teeth in their oral cavity. Subjects in the mothers group were aged 27–40 years, were non-smokers, and had 28 teeth excluding the third molars. Participants in mothers group were subjected to a baseline oral examination consisting of an inspection of dental caries, probing depths and clinical attachment loss, and a determination of the gingival index and the plaque index. They were found to have no dental caries or periodontal disease (overall clinical attachment loss of <1 mm). All participants were examined clinically by a single calibrated examiner before sampling.

**Table 1:** Demographic and clinical characteristics of study subjects.

	**Caries-free (*n* = 15)**	**Caries (*n* = 16)**	**Mother (*n* = 15)**
Mean age	56.8 ± 8.50 (months, mean ± SD)	57.38 ± 8.53 (months, mean ± SD)	32.8 ± 3.43 (years, mean ± SD)
No. (%) male	9 (60%)	4 (25%)	0 (0)
No. (%) female	6 (40%)	12 (75%)	15(100%)
DMFT (mean ± SD)	0	4.38 ± 2.32	0.27 ± 0.59
DMFS (mean ± SD)	0	7.88 ± 2.15	0.47 ± 1.13

*DMFT, number of decayed, missing and filled teeth*.

*DMFS, decayed, missing and filled tooth surfaces*.

All participants in our study met the following inclusion criteria. They were all generally healthy and reported no antibiotic intake in the previous 3 months. The participants had not received any dental treatments (such as dental fillings, root canal therapy, fluoride application or ultrasonic teeth cleaning) in the preceding 3 months. Subjects with systematic diseases were excluded. In addition, a questionnaire assessing potential risk factors for ECC was completed by parents or guardians before sample collection. The questions involved topics, such as demographic characteristics of parents/guardians (name, gender, age, educational level and family income) and of the children (name, gender, age, birth date and monozygoticity/dizygoticity), oral hygiene habits of the children (age at which tooth brushing started, frequency of brushing, whether supervision was needed and whether fluoride toothpaste was applied when tooth brushing), and eating habits, especially concerning sweets consumption and feeding practices during the infant period (breastfeeding or bottle feeding in the first 3 months).

### Sample collection

Supragingival plaque samples were collected at 9:00 a.m. and 15:00 p.m. and all participants were instructed not to brush their teeth on the day of sample collection. In addition, all subjects in our study refrained from eating or drinking 1 h prior to sample collection. Caries group samples were pooled from the lingual and proximal surfaces of decayed teeth were pooled and samples from the dental plaque in caries lesions were pooled. Samples from the caries-free group and the mothers group were collected from the labial, lingual and proximal surfaces of healthy teeth (including anterior and posterior teeth). All supragingival plaque samples were obtained by scraping the tooth surfaces with a sterile metal excavator. The metal excavator was immersed in a sterile 1.5 ml eppendorf tube containing 1 ml of Ringer's solution. The samples were transported on ice to the laboratory, and stored at −20°C until genomic DNA extraction.

### Genomic DNA isolation and amplification

Bacterial DNA was extracted from supragingival plaque samples using the modified phenol/chloroform/isoamyl alcohol (PCI) procedure. A full description of the genomic DNA extraction procedure is provided in the Supplementary Materials section. Total DNA was quantified with a Nanodrop spectrophotometer (NanoDrop Technologies, Wilmington, DE, USA), and genomic DNA was stored at −80°C until further use.

The genomic DNA was sent to the MIM Core Facility at the Forsyth Institute (Cambridge, MA, USA). The HOMINGS laboratory procedures were carried out according to the specifications of a modified protocol. Briefly, 16S rRNA amplicons were generated with the following primers: V3-V4 forward (341F): AATGATACGGCGACCACCGAGATCTACACTATGGTAATTGTCCTACGGGAGGCAGCAG and reverse (806R): CAAGCAGAAGACGGCATACGAGATNNNNNNNNNNNNAGTCAGTCAGCCGGACTACHVGGGTWTCTAAT. Then, 10–50 ng of DNA was used for PCR-amplification, followed by purification using AMPure beads. The cycling conditions were as follows: initial denaturation at 94°C for 3 min, 35 cycles of denaturation at 94°C for 45 s, annealing at 50°C for 1 min, elongation at 72°C for 1.5 min, and a final extension step at 72°C for 10 min. PCR samples were then purified using AMPure beads (Beckman Coulter Genomics, Danvers, MA). One hundred nanograms of each library was pooled, gel purified, and quantified using a bioanalyzer and subsequently with quantitative PCR (Light Cycler 96 Real-Time PCR System; Roche Diagnostics GmbH, Mannheim, Germany). Twelve picomolars of the library mixture spiked with 20% PhiX was run on a MiSeq (Illumina, San Diego, CA). In this study an average of 49,862 sequences, with ~441 bp per sequence were obtained.

### Accession numbers

Raw sequences generated from this study were deposited into the NCBI Sequence Read Archive under the accession number SRP126050.

## Results

### HOMINGS general findings

A total of 46 supragingival plaque samples were analyzed, 16 of which belonged to children with dental caries, 15 to caries-free children and 15 to mothers who were orally healthy. On average, the number of sequences each sample generated was 49,862 (range 26,082–72,141), out of which 51.0% (range 30.9–71.6%) and 12.3% (range 7.9–20.8%) were identified at the species-level and genus-level, respectively. In addition, an average of 36.8% (range 19.9–57.9%) of the generated sequences could not be assigned to either a species-specific or genus-specific probe sequence based on blast against the probeseq database. The number of positive identification targets reached 406 (342 identified at the species-level and 64 identified at the bacterial genus level), corresponding to 53% of the total 768 probe sequences present in the probeseq database. The average number of positive identifications was 174 (range 130–255) in whole samples among three groups. Significantly more targets were identified in samples from the mothers group (mean 201, range 166–255) than in samples from caries (mean 159, range 130–193) and caries-free (mean 162, range 139–188) groups (*P* < 0.05). However, there was no significant difference between caries and the caries-free groups (*P* > 0.05). At species level, HOMINGS identified of 342 oral bacterial taxa in all samples. We compared the relative abundance identified at the species level in the caries and caries-free groups using the Mann-Whitney *U*-test, and Benjamini-Hochberg correction was used to control for multiple comparisons, and an adjusted *P*-value <0.0001 was considered statistically significant. The results indicate that the relative abundance of *Streptococcus mutans, Lactobacillus fermentum, Neisseria sica*, and *Veilonella dispar* was much higher in the caries group (see Table 2).

**Table 2:** The species identified with significantly different relative abundance in samples from individuals with caries and caries-free groups.

**Species**	**Relative abundance (%)**			
	**Caries (*n* = 16)**	**Healthy (*n* = 15)**	**Raw *p*-value**	**Adjusted *p*-value**
*Actinomyces israelii*	1.32783	2.14795	7.37 × 10^−8^	2.43 × 10^−6^
*Capnocytophaga_sputigena*	0.00321	0.35721	5.74 × 10^−8^	1.32 × 10^−6^
*Fusobacterium periodonticum*	0.47639	1.73852	3.58 × 10^−8^	6.03 × 10^−6^
*Lactobacillus fermentum*	0.00674	0.00035	2.06 × 10^−7^	1.64 × 10^−5^
*Porphyromonas sp ot 279*	0.38653	2.57396	5.83 × 10^−7^	7.57 × 10^−5^
*Streptococcus mutans*	0.00547	0.00052	8.58 × 10^−7^	6.44 × 10^−5^
*Neisseria sicca*	0.00267	0.00036	1.73 × 10^−6^	6.32 × 10^−5^
*Haemophilus parainfluenza*	1.30546	2.33471	2.23 × 10^−6^	5.38 × 10^−5^
*Veillonella diapar*	0.11468	0.00893	5.76 × 10^−6^	6.01 × 10^−4^
*Leptotrichia sp ot 498*	0.00486	0.72474	1.47 × 10^−5^	5.06 × 10^−4^
*Actinomyces massiliensis*	0.46535	0.00365	4.76 × 10^−5^	3.53 × 10^−4^
*Prevotella oulorum*	0.00156	0.00855	4.83 × 10^−^	6.87 × 10^−4^

*P-value <0.0001 was considered statistically significant*.

### 16S rRNA sequences analysis and statistical analysis

The input included a total of 9,742,669 raw pyrosequencing reads. Raw sequence data were quality-checked (denoised) with Ampliconnoise (Schloss et al., 2013) and chimeras removed with UCHIME (Edgar et al., 2011) using default settings. After quality checks, all analyses were performed using QIIME (V.1.9.0) software (Caporaso et al., 2010). High quality sequences were clustered into OTUs at 97% pairwise identity (OTU_0.03_) using the USEARCH (version 9.0) algorithm (Edgar, 2013) with default settings, and representative sequences from each OTU_0.03_ were aligned against the Greengenes (version 13-8) reference alignment (Desantis et al., 2006) using PyNAST (Caporaso et al., 2010). Aligned sequences were then used to build phylogenetic trees using the Fast Tree method (Price et al., 2009).

Taxonomy assignment of each representative sequence was implemented using the BLAST algorithm against the Silva108 curated database (Pruesse et al., 2007). Sequences with reference sequence hit below 90% were called unclassified.

Alpha diversity of OTU libraries was described using the Chao1, Shannon, Simoson, Coverage, Observed species and ACE metrics as implemented in QIIME. Evenness was described by the Gini coefficient (Gcorr.), by calculating the area under the Lorenz curves per OTU_0.03_ pair (Wittebolle et al., 2009) with correction to minimize bias (Edwards et al., 2011). Alpha diversities between two communities were compared using a Student's *t*-test in R software (Knezevic et al., 2007). Distance matrices were constructed using the Unweighted and Weighted UniFrac algorithms in QIIME from the whole community phylogenetic tree. For statistical testing of sample groupings in a distance matrix, we applied ANOSIM with *P*-value <0.05 as significant. Significance of beta diversity for spatial heterogeneity of microbial communities was calculated with the algorithm described in elsewhere (Gülay and Smets, 2015) in combination with Weighted UniFrac diversity metric. Furthermore, in order to analysis the connection of microbiota and geographic characteristics, beta diversity was analyzed using both Bray-Curtis and Jaccard in Vegan package (https://cran.r-project.org/web/packages/vegan/index.html). The LEfSe analysis (http://huttenhower.sph.havard.edu/galaxy) are used to find metagenomic biomaker and explanation in different groups. The figure illustrates in detail the for-mat of the input (a matrix with *n* rows and m columns) and the three steps performed by the computational tool: the KW rank sum test on classes, the pairwise Wilcoxon test between subclasses of different classes, and the LDA on the relevant features. PICRUSt is a bioinformatics software package designed to predict metagenome functional content from 16S rRNA survey (http://picrust.github.io/picrust). PICRUST (Huttenhower Lab, V 1.0.0) was used to recaptures key findings from KEGG and accurately predict the abundance of bacterial gene family in host associated communities. STAMP software package (v 2.1.3) were used to for analyzing taxonomic or metabolic profiles that promotes “best practices” in choosing appropriate statistical techniques and reporting results. The metabolic activities differences between different groups were evaluated using the Wilcoxon rank-sum analysis or Turkey-Kramer analysis of the variance (ANOVA) test, and with *P*-value <0.05 as significant.

#### Distribution of OTUs in all samples

**Figure 1:**
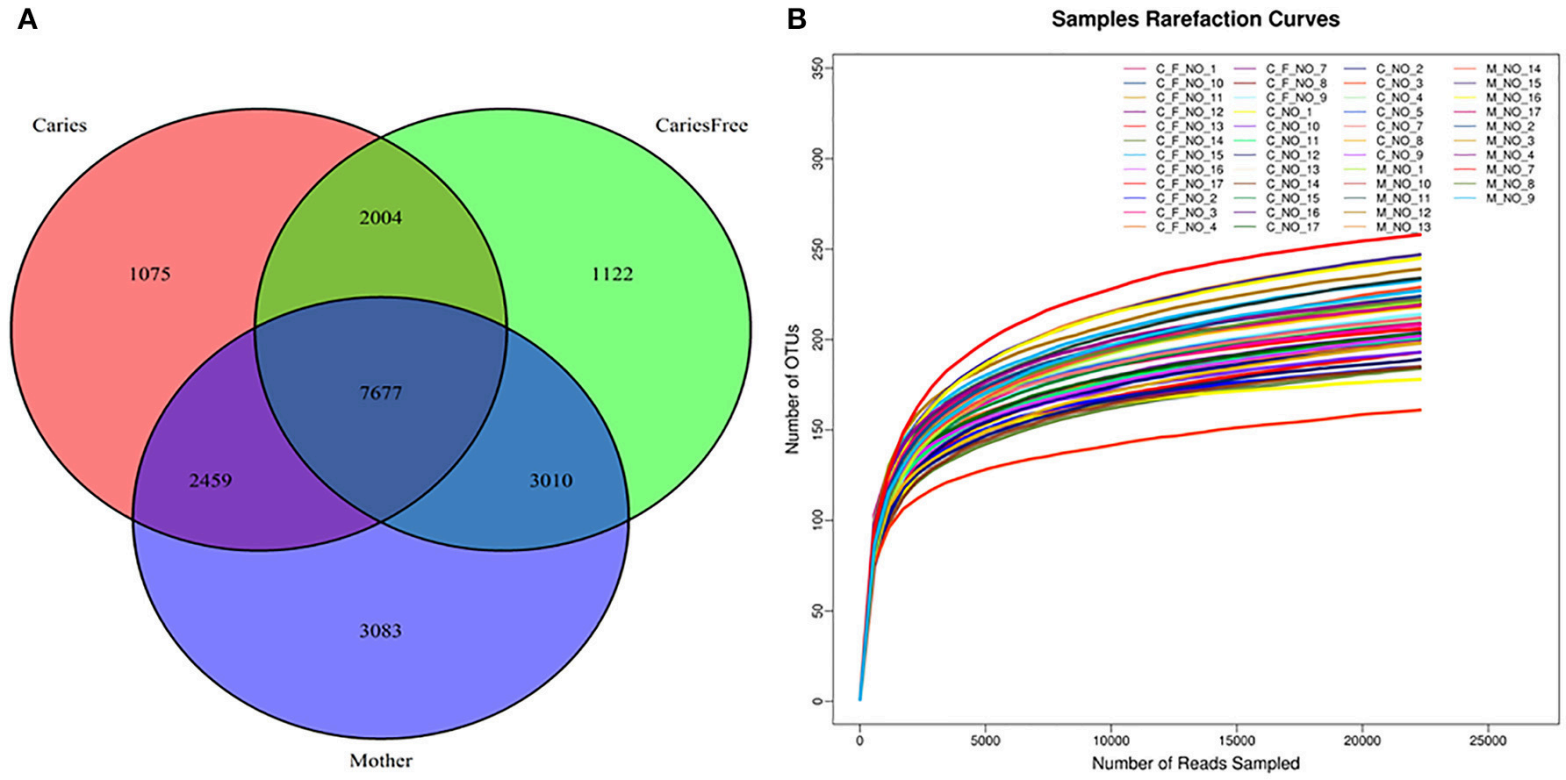
**(A)** Venn Diagram represented number of OTUs among three groups after rarefaction to 22,841 sequences per individual. Red circle showed the Caries group; mother group was the blue circle; Green circle represented the caries-free group. The numbers in each circle showed the number of OTUs were found in each group, and overlap represented the shared OTUs in different groups. **(B)** Rarefaction curves for individuals in caries, caries-free and mother group. Rarefaction curves generated from the phylogenic distances showed that the sequencing depth of the samples in our study was enough to proceed with the next step in the analysis.

A total of 20,430 operational taxonomic units (OTUs) were identified; the OTU abundance in each group is shown in Figure 1A. A total of 13,215 OTUs were identified in the caries group, 13,813 in caries-free group and 16,229 OTUs in the mothers group. Considering mothers group samples together, a comparable number of supragingival plaque microbe OTUs were shared with caries (62.5%) and caries-free group (65.9%). There was also a large percentage of overlap between caries and caries-free group communities.

#### Microbial alpha diversity in the three groups

**Figure 2:**
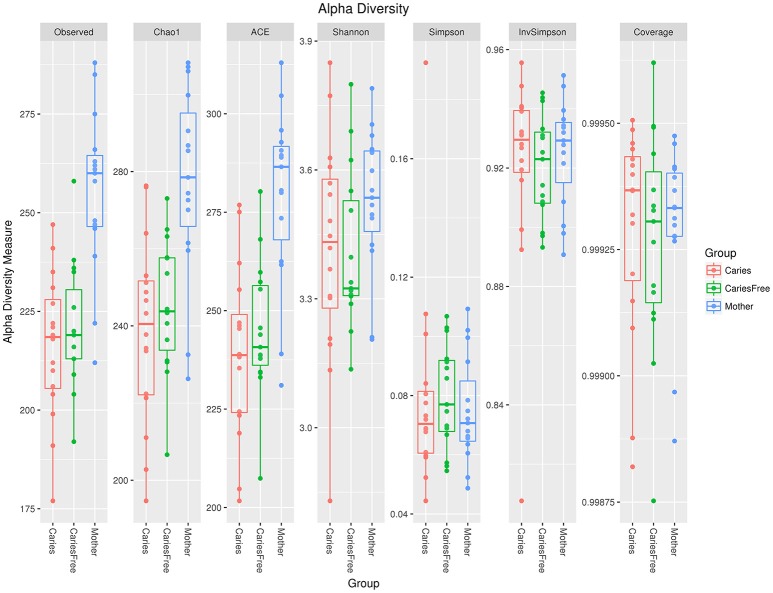
Alpha diversity indices in three groups. There were significant differences in microbial richness between the mothers group and the caries (*P* < 0.001) and caries-free (*P* = 0.001) groups. The Shannon index in the mothers group was much higher than in the caries (*P* = 0.003) and the caries-free (*P* = 0.009) groups.

Alpha diversity analysis was performed after rarefaction to 22,841 sequences per sample. Diversity indices were used to evaluate the richness and evenness of microbial profiles. Rarefaction curves generated from the phylogenic distances show that the sequencing depth of the samples in our study was enough to proceed with the next step in analysis (see Figure 1B). The alpha diversity was measured as the number of observed species and Shannon index (see Figure 2). In terms of the number of observed species in each group, there were significant differences in microbial richness between the mothers group and the caries group (*P* < 0.001) and the caries-free group (*P* = 0.001). The Shannon index in mothers group was much higher than in caries (*P* = 0.003) and caries-free (*P* = 0.009) group. In contrast, both the numbers of observed species and Shannon indices showed no significant differences between caries and caries-free group (*P* > 0.05). Based on the analysis of alpha diversity, we conclude that the richness of the microbial composition was relatively higher in mothers group, while there was no significant differences between caries and caries-free groups.

#### Taxonomy analysis and comparison between three groups

**Figure 3:**
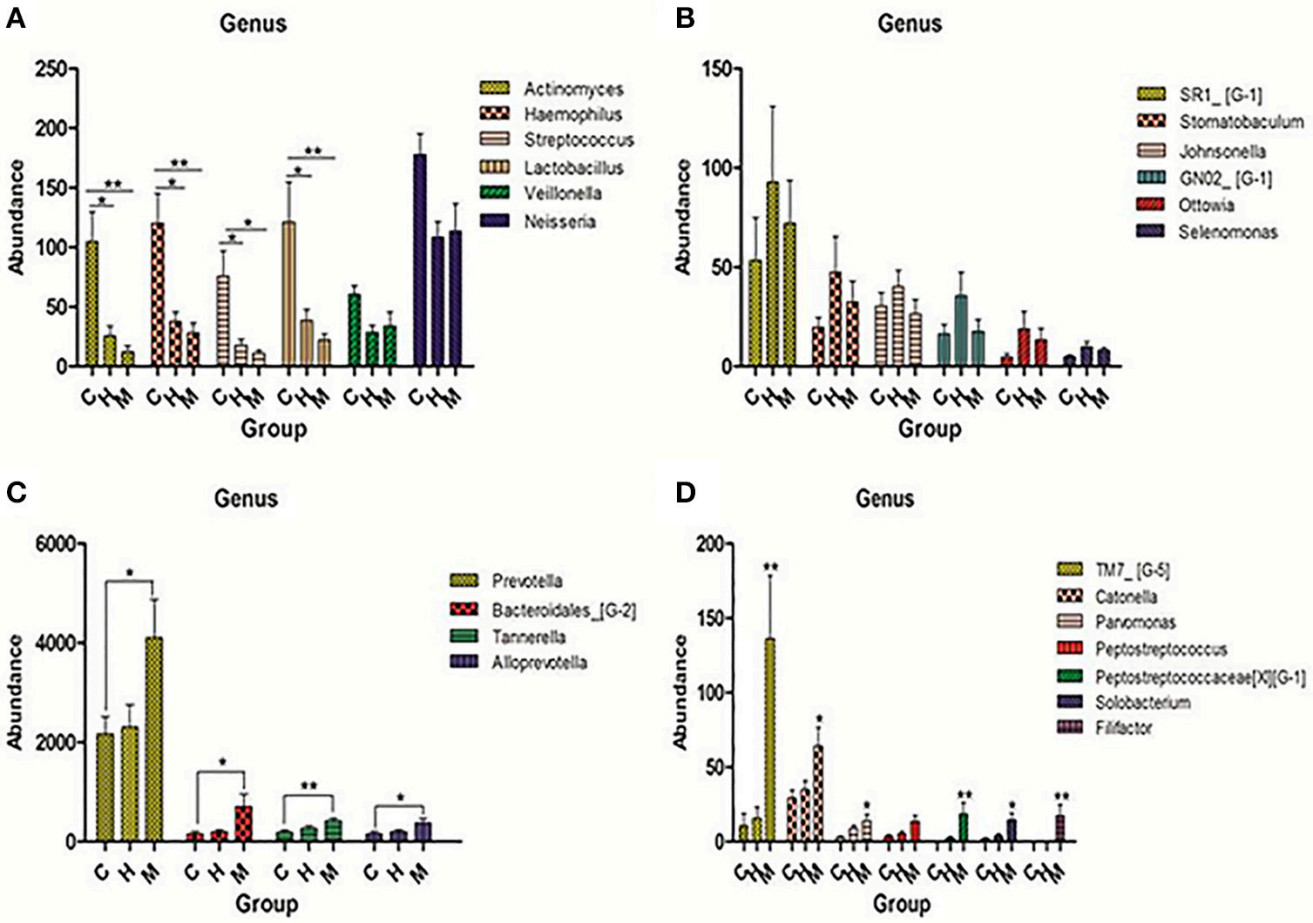
Pick out 50 genera which were ranked top in the total genera and then analyzed the predominant members in each group. **(A)** Genera were abundant in caries group. **(B)** Genera were abundant in caries-free group. **(C,D)** Genera were abundant in mother group. The statistical methods of one-way ANOVA followed by Turkey's multiple comparison *post-hoc* tests were used in **(A,C)** and Kruskal-Wallis tests were used in **(D)** (*P < 0.1,^**^P < 0.05, ^***^P < 0.01 mean ± s.e.m).

Comparing the filtered reads with the reference sequences of Greengene (version 13-8), we found that over 90% of the bacterial diversity in each sample was contributed by the following five phyla: *Fusobacterium* (44.8%), *Bacteroidetes* (21.7%), *Actinobacteria* (19.4%), *Proteobacteria* (4.9%), and *Firmicutes* (3.1%). A total of 116 genera were identified. The following six predominant genera were detected in supragingival plaque microbiota were six: *Leptotrichia* (30.0%), *Fusobacterium* (14.7%), *Corynebacterium* (10.1%), *Prevotella* (7.3%), *Capnocytophaga* (5.7%), and *Actinomyces* (5.4%). However, the relative abundance of these genera in each sample was different. Furthermore, we selected 50 genera which ranked at the top of the total genera and analyzed the predominant members in each group. Comparing the relative abundance in each group, we found that *Lactobacillus, Haemophilus, Streptococcus*, and *Actinomyces* were present at a higher level in caries group than caries-free and mothers group (*P* < 0.05; see Figure 3A). Rich abundant genera in caries-free group were displayed (see Figure 3B). In mothers group the predominant genera included *Bacteroidales, Catonella, Parvimonas, Prevotella*, and *Tannerella* (see Figures 3C,D).

#### Hierarchical cluster analyses

**Figure 4:**
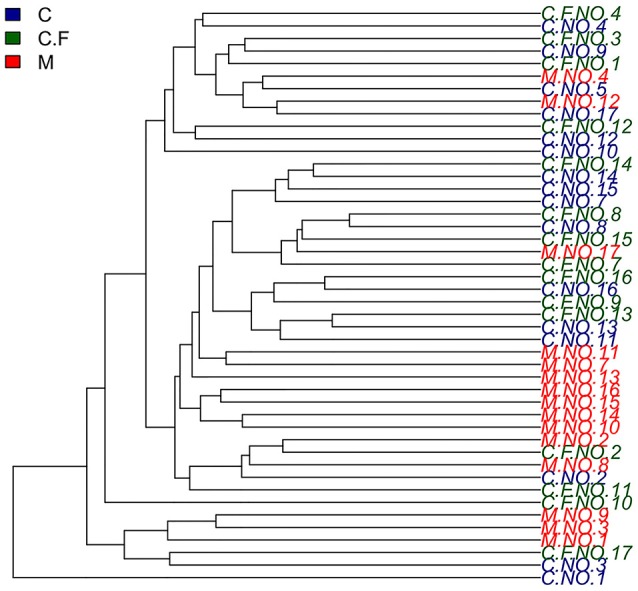
Hierarchical cluster Analyses shows a similarity plot based on OTUs table for all individuals using average and vegan as a cluster method. In this study twins number 4, 8, 12, 13, 14, and 16 were monozygotic twins.

To analyze the similarities of the bacterial community profiles of all individuals, data from the OTUs table were analyzed by cluster analysis using vegan packages in R software. Data from all samples were analyzed together in this analysis. Interestingly, all of the monozygotic twins analyzed in this study exhibited a higher degree of similarity than dizygotic twins, in terms of the composition of the bacterial communities (see Figure 4). Based on these results, we compared the weighted UniFrac distances between monozygotic and dizygotic twins. The results confirmed that monozygotic twins shared a higher degree of similarity than dizygotic twins (*P* = 0.011).

#### Microbial beta diversity in the three groups

**Figure 5:**
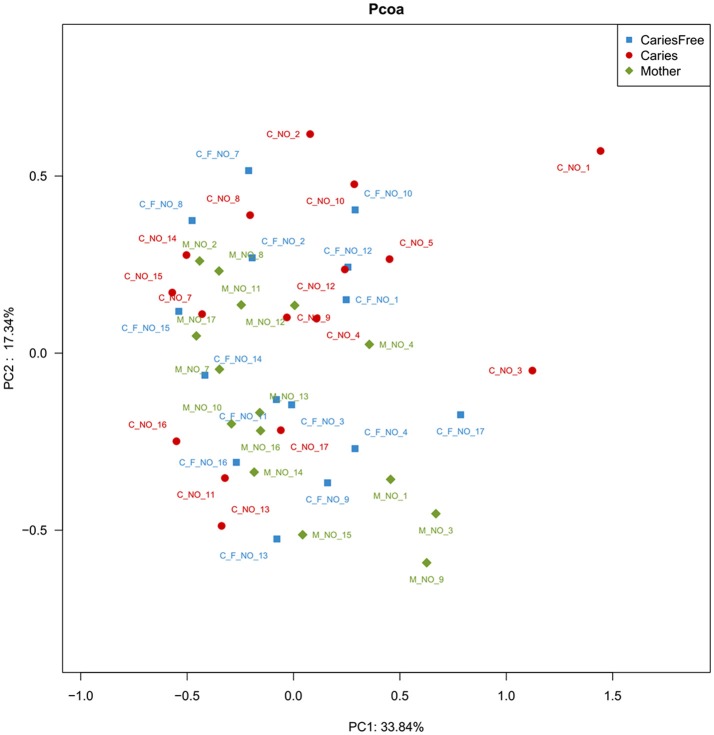
PCoA analysis of the supragingival plaque bacterial communities. The analysis was based on the Bray-Curtis similarity matrix constructed using the square-root-transformed OTU. The percent of variation explained is given behind of the value of PC.

To further compare the bacterial community diversity of the three groups, we performed a principal coordinate analysis of the Weighted UniFrac beta diversity matrix from the whole community phylogenetic tree (see Figure 5). The first three principal coordinates explained 33.84, 17.34, and 15.28% of the variation, respectively. However, we found no significant differences between the three groups based on the analysis, moreover separation between the populations of three groups was not obvious. These results indicated that the supragingival plaque microbial composition was similar, according to the Weighted UniFrac beta diversity matrix.

**Figure 6:**
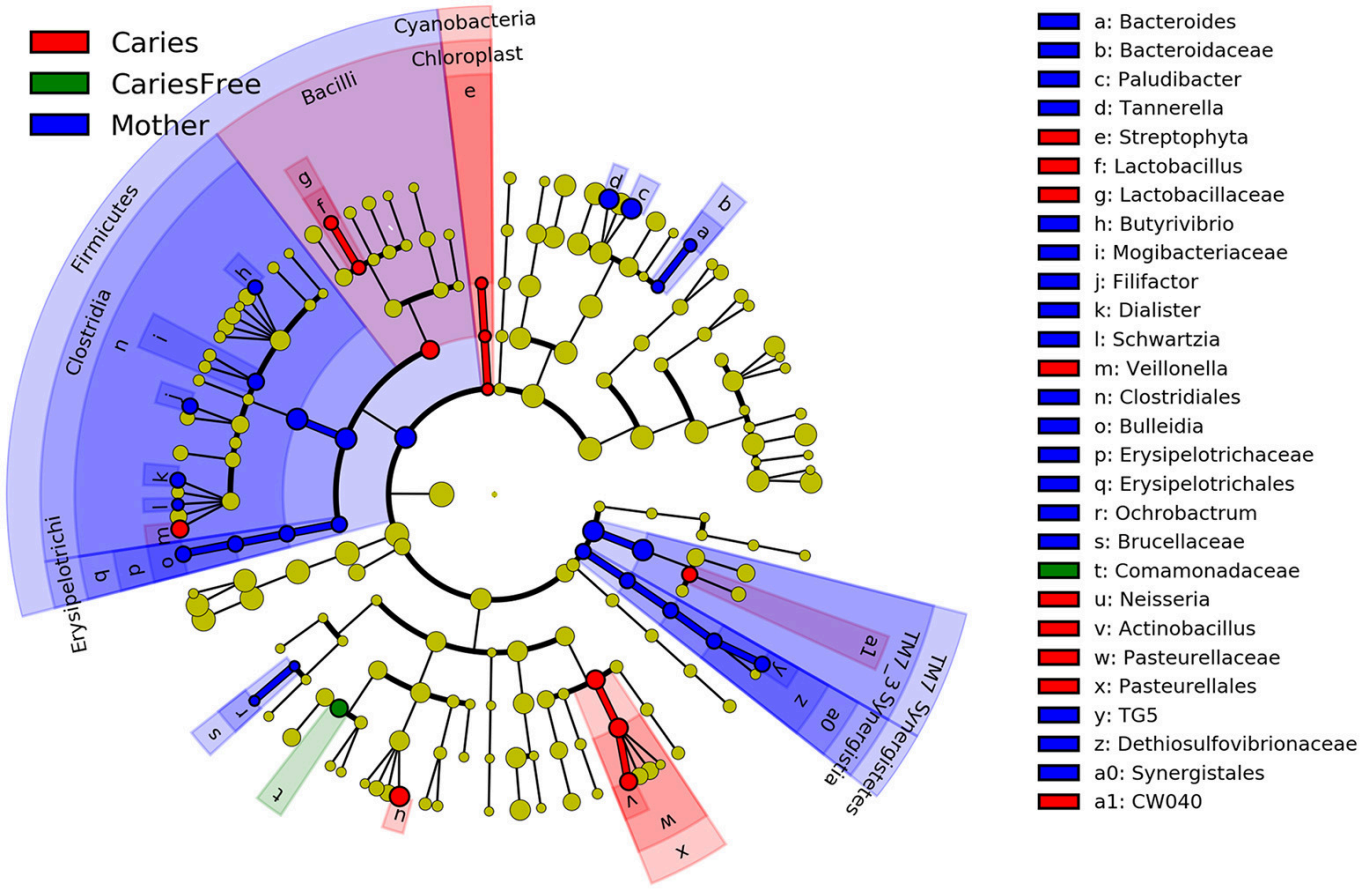
LEfSe cladogram identified the most differentially abundant taxons in three groups. Taxonomic cladogram obtained from LEfSe analysis of 16S amplicon sequences (relative abundance ≥0.5%). (Blue) mother group-enriched taxa; (Red) taxa enriched in caries group; (Green) showed the enriched taxa in caries-free group. The brightness of each dot is proportional to its effect size.

Furthermore, LefSe analysis was used to analyze and compare enriched taxa in each group (see Figure 6). Individuals with caries tended to show a higher relative abundance of *Lactobacillus, Veillonella, Neisseria* and *Actinobacillus*. Moreover, these results are similar to those obtained from our analysis of the predominant genera in the caries group, suggesting that the increased abundance of these taxa may be an indicator of the occurrence of ECC. Furthermore, the mothers group had the most enriched taxa among three groups, whereas only one taxon was enriched in the caries-free group, and these results are consistent with the previous analysis of alpha diversity, indicating a higher degree of species richness in mothers group.

#### Metabolic activities analysis

**Figure 7:**
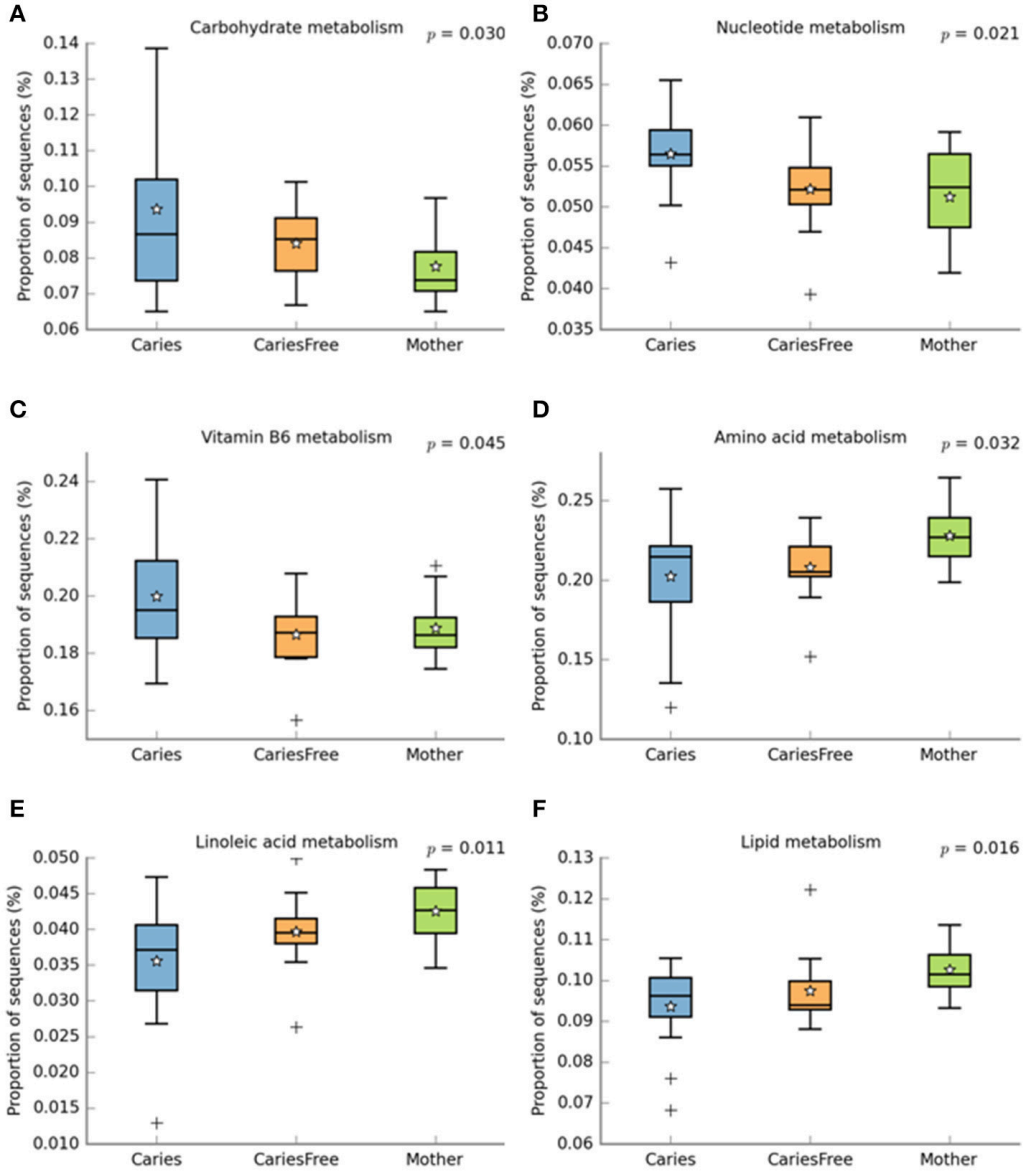
PICRUST analysis to predict metagenome functional content from 16S rRNA surveys, then use STAMP software package for analyzing metabolic profiles that promote “best practices.” Using STAMP to identify metabolic activities which are differentially abundant between different groups. **(A–C)** shows metabolic activities which are abundant in caries children; **(D–F)** represent metabolic activities which are abundant in mother group. + shows the presence of outliers.

Phylogenetic Investigation of Communities by Reconstruction of Unobserved States (PICRUSt) is a bioinformatics software package designed to predict metabolic functions from marker genes, such as 16S rRNA. Based on the bacterial profile composition obtained with 16S sequencing, we used PICRUSt and Stamp analysis to predict differences in metabolic function in different groups (see Figure 7). The results indicate that nucleotide metabolism, vitamin B6 metabolism, and carbohydrate metabolism are significantly associated with caries, and the results suggest that *Streptococcus mutans, Lactobacillus fermentum, Actinomyces israelii*, and *Neisseria sica* are involved in these metabolic activities. Furthermore, amino acid metabolism, lipoic acid metabolism and lipid metabolism were predicted to be influenced by the microbiota to a higher extent in the mothers group.

To further examine the effects of environmental demographic characteristics on the supragingival plaque microbiome construction, the permutational multivariate analysis of variance using distance matrices (PERMANOVA) and a multivariate analog of Levine's test for homogeneity of group dispersions (BETADISPER) were conducted. Comparing the clinical and demographic characteristics of children in the caries group, we found that gender and certain daily eating habits are correlated with ECC (see Table 3). Daily eating habits were categorized as (i) eating sweet food more than three times per day and (ii) eating sweet food equal or less than one time a day. Among the 16 children from the caries group included in the final analysis, most children were female (75%), with 25% being male. The PERMANOVA partitions the variability based on Bray-Curtis dissimilarity matrix. The results indicate that there was no significant difference in supragingival plaque microbiome community between different genders (*P* > 0.05). Eating habits like the frequency of taking sweet foods were found to be associated with ECC (*P* < 0.05). In order to further examine whether the two eating habits (eating sweet food more than three times a day and eating sweet food equal or less than one time a day) correlated with the microbiome composition, the PERMONOVA and BETADISPER tests based on the Bray-Curtis dissimilarity matrix were conducted to explore the difference between the microbiome communities. The results show a significant difference between the “eat sweet food more than three times” day and the “eat sweet food equal or less than one time a day” categories (*F* = 30.46, *R*^2^ = 0.12, *P* < 0.001). The BETADISPER showed that the average distance between children who take sweet food more than three times a day was 0.64, and it was 0.26 between children eat sweet food equal or less than one time a day.

**Table 3:** Environmental factors related with ECC.

		***n***	**Percent (%)**
Gender	Male	4	25
	Female	12	75
Frequency of taking sweet food	*n* > 3	13	81.25
	*n* ≤ 1	3	18.75
Age of beginning toothbrush	≥2 (years)	9	56.25
	<2 (years)	7	43.75

## Discussion

ECC represents a great and growing concern for preschool children. Increasing evidence suggests that a combination of environmental factors, such as diet, oral hygiene and behavioral habits, as well as host genetic factors and the composition of the oral microbiota, are important for the initiation and development of dental caries (Harris et al., 2004; Petersen, 2010). Assessment of the probable risk factors of developing caries is important for caries prevention and management. Furthermore, analyzing the possible contribution of oral microorganisms to dental caries is complicated due to the variability of the oral microbiota. The subjects in our study were twin pairs with different caries phenotypes, where one twin had ECC while the other one was healthy. In our study, Human Oral Microbe Identification using Next Generation Sequencing (HOMINGS) was applied to analyze the composition and diversity of the oral microbiome in supragingival dental plaque samples obtained from 14 twin pairs, a set of triplets and their mothers. Higher levels of abundance of a combination of certain oral microorganisms, such as *Streptococcus mutans, Lactobacillus fermentum, Actinomyces israelii, Neisseria sica*, and *Veillonella dispar* at higher levels of abundance, can increase the risk for ECC. Moreover, since five pairs of twins plus the set of triplets were monozygotic, while the rest were dizygotic, comparison of the weighted UniFrac distances between monozygotic and dizygotic twins allowed us to investigate the impact of host genetics on the composition of oral microbiota.

Dental caries is considered as the most prevalent and multifactorial oral cavity disease in preschool children (Islam et al., 2007). It is known that teeth, cariogenic bacteria and fermentable sugars are the major factors influencing the development of this disease. Under normal circumstances, the oral microbiota plays an important role in maintaining the homeostasis of oral cavity. A previous study showed that the bacterial composition of supragingival dental plaques was dramatically different when samples were taken from healthy tooth surfaces, white spots orcavities (Gross et al., 2010). This observation suggested that an increase in the frequency of various cariogenic bacteria might lead to the development of ECC. In our attempts to obtain comprehensive bacterial profiles of supragingival plaques in children aged 3–6 years ole children, our findings revealed new insights in the composition of the children's supragingival plaque microbiomes. Most importantly, the bacteria related with ECC were identified at the species level in our study. *Streptococcus mutans* is the leading cause of tooth decay and was considered as the most cariogenic *Streptococcus* species in previous studies (Ajdić et al., 2002; Alaluusua and Renkonen, 2010; Buischi et al., 2010). Although *Streptococcus* was not as abundant as *Actinomyces* in our study, *Streptococcus mutans* exhibited a much higher frequency in carious children. These results suggest that *Streptococcus mutans* is associated with ECC. Previous studies have revealed that preschool children harboring both *Streptococcus mutans* and *Streptococcus sobrious* show a higher incidence of ECC (Okada et al., 2005). Furthermore, our results suggest that found that *Lactobacillis fermentum* is associated with ECC, and it is consistent with a previous study that found that *Lactobacillus* were involved in dental caries (Smith et al., 2001; Piwat et al., 2010; Teanpaisan et al., 2011). This supports the notion that caries develop when multiple oral microorganisms act collectively (Jenkinson and Lamont, 2005; Kuramitsu et al., 2007). There are three major hypotheses that attempt to explain the etiology of dental caries: the specific plaque theory, the non-specific plaque hypothesis and the ecological plaque hypothesis (Theilade, 1986; Loesche, 1992; Marsh, 1994). A report by Kuramitsuet has shown that dental caries may be caused by a complex microbial community rather than a single pathogen (Kuramitsu et al., 2007). Our results support the ecological plaque hypothesis, which states that pathogenesis of caries connotes a microbial environmental shift toward a more acidogenic and aciduric microflora in dental plaques, due to frequent carbohydrate intake (Andreadis and Kalfas, 2014). We observed an increase in the number of cariogenic bacteria, e.g., *Streptococcus mutans, Lactobacillus fermentum, Neisseria sica*, and *Veilonella dispar* in carious children, which suggests that the oral microecological balance had been disturbed in these children. The result is consistent with previous studies that suggested that oral microbial homeostasis is partly a result of dynamic balance of both synergistic and antagonistic microbial interactions (Marsh, 2006). Once homeostasis is disturbed, leading to a shift in the balance of oral microbiome, this predisposes the individual to conditions like tooth decay and periodontal diseases (Burne and Marquis, 2000).

Moreover, comparison of the microbial profiles of the mothers group with the (caries and caries-free) children groups, showed that *Prevotella, Tannerella*, and *Bacteroidales* were much more abundant in the mothers, whereas the relative abundance of these genera was very low in the caries and caries-free groups. This suggests that the composition of the oral microbiome may change in different periods of life, e.g., during the stages of deciduous and permanent dentition. Our results support the previous findings of Cirelaard who noted that deciduous teeth harbor a higher proportion of *Proteobacteria* than *Bacteroidetes*, while *Spirochaetes* and the candidate division *TM7* increase with increasing age, reflecting the maturation of the microbiome, driven by biological changes associated with age (Crielaard et al., 2011). In addition, a study by Ling, who analyzed the overall taxonomic distribution of metagenomic data, showed that the diversity of the salivary microbiota in children was more complex than in adults (Ling et al., 2013). In this regard, the question arises as to why and how the microbiome changes with increasing age, and intensive studies will be needed in the future to answer these questions. In our study, cariogenic bacterial transmission from mother to child was not obvious. This finding was similar with a report which described that microbial genotypes found in children that are different from those derived from their mothers (Mattos-Graner et al., 2001). This indicates that an additional source of cariogenic microbial transmission might exist. Our result is not consistent with former studies, which confirmed that microorganisms considered to be associated with the development of caries are transferred by saliva from people in the child's closest environment (Mattos-Graner et al., 2001; Cephas et al., 2011). Those data indicated that oral colonization by *S. mutans* usually happens by contact with the most closely related person whose oral cavity is colonized by such microorganisms (Dominguezbello et al., 2010). Moreover, “vertical transmission,” cariogenic bacteria occurs in ~60% of infants when the abundance of microorganisms in the mother's saliva amounts to 10^5^ or more colony forming units per milliliter of saliva (CFU/ml) compared to 6% of infants when the abundance in the mother's saliva is 10^3^ CFU/ml of saliva (Berkowitz, 2006; Struzycka, 2014). One potential reason behind the discrepancy between these studies and the present study is that, in our study, individuals in the mothers group were all orally healthy, and no carious teeth were detected in this group. Moreover, whereas all of our child study subjects were at least 3 years old, the transmission of caries-related bacteria like *S. mutans* and *Lactobacillus* occurs early in life; the transmission of *S. mutans* from mother to child has been reported to occur prior to dental eruption, usually at 3 months of age (Wan et al., 2001).

Third, we have further shown that monozygotic twin pairs shared a higher degree of similarity in the composition of their oral bacteria than dizygotic twins, suggesting that host genetic factors play a role in determining the oral bacterial composition. It is well-known that twins offer a precious opportunity to untangle the influence of genes and the environment-of nature and nurture (O'Brien, 2000). Monozygotic twins come from a single fertilized egg that splits into two, so they share essentially the same genetic information (Fraga et al., 2005; Reyes et al., 2010); any difference between the twins, one having caries for example must be due to environmental factors. Moreover, by comparing the experiences of monozygotic twins with those of dizygotic twins (Tsai and Wu, 1988; Kaminsky et al., 2009), who come from separate eggs and share on average half of their DNA, we can quantify the extent to which our genes affect our lives. If monozygotic twins are more similar to each other with respect to a certain diseases than dizygotic twins are, then vulnerability to the disease must be rooted at least in part in heredity (Reyes et al., 2010). These are two kinds of twin model research–comparing the differences between monozygotic twins to point out the influence of the environment, and comparing monozygotic twins with dizygotic twins to measure the role of inheritance–and both have been proven important in the development of our understanding of the interplay of nature and nurture in determining the vulnerability to certain diseases (Hammond et al., 2000; Selmi et al., 2004; Reyes et al., 2010). In the present study, we compared the weighted UniFrac distances between monozygotic and dizygotic twins. Our results indicate that monozygotic twins share a higher degree of similarity than dizygotic twins in microbiome composition. We also compared six monozygotic twin pairs and several environmental factors using a questionnaire survey, but we did not find any significant difference in monozygotic twin pairs. One possible explanation for this is the fact that the number of monozygotic twins in our study was small. Moreover, each monozygotic twin pair lived in the same household and shared many similarities in daily life. Another factor is the fact that this is a cross-sectional study, while time is a crucial factor in the development of caries. The monozygotic twins showed differing caries phenotypes at the moment of our study, but it is not clear that whether this difference would remain in the future. Therefore, a greater sample size and longitudinal research are required to fully determine the role of hereditary factors in the development of caries. At the same time, dietary factors, such as the frequency of eating sweet foods, can change the microbial composition, as confirmed in our study. Combining the above results, we can conclude that environmental factors can change the abundance of certain oral bacteria, especially cariogenic bacteria, consistent with previous surveys (Wang et al., 2012; Lorber et al., 2014; Abbasoglu et al., 2015). However, previous studies with twins have reported a high degree of concordance as regards the dental caries phenotype in monozygotic twins. In 1979, a study involving 100 monozygotic and 120 dizygotic twin pairs, showed that genetic factors influenced the occurrence of dental caries, both in primary and permanent dentition (Fairpo, 1979). Similar results were found by Bretz, who studied 388 twin pairs in 2005 and estimated the importance of hereditary factors in dental caries by measuring the percentage of phenotypic variance, demonstrating that hereditary factors accounted for 70% of the dental caries phenotype (Bretz et al., 2005). In contrast, in our study monozygotic twins shared the same bacterial profile yet they exhibited different caries phenotypes. A possible explanation for the difference between these results is the fact that caries develop over time. The most widely accepted hypothesis regarding the pathogenesis of caries is that it is the result of the combination and interaction of four major factors: biofilm, diet, time, and host (Keyes, 1960; Evans et al., 2010). Since we conducted a cohort study, not a longitudinal study, we can only conclude that monozygotic twin pairs showed discordant caries phenotypes at the specific moment of sample collection. However, it is unclear whether these monozygotic twin pairs will still show the discordant caries phenotypes months or years later.

## Conclusions

In summary, we conclude that a complex microbial community structure can be found in supragingival plaques. *Streptococcus mutans, Lactobacillus fermentum, Actinomyces islaelii, Neisseria sica*, and *Veilonella dispar* are positively correlated with ECC, and the richness of the microbiota community may increase with age. However, how and why this change occurs is unclear, so further studies and analyses are required to address this question. The composition of the supragingival plaque bacterial profile is more similar in monozygotic twin pairs than in dizygotic twins, suggesting that genetic components influence the oral microbiome composition. At the same time, environmental and behavioral factors, such as the frequency of eating sweet foods have an impact on the distribution of caries-related bacteria. The results of our study will provide a reference for future research on the etiology of ECC and help clinicians implement adequate preventive strategies against ECC.

## Author contributions

MD and HJ conceptualized the study and provided expert advice in analysis and evaluation of the manuscript. YZ and MZ planned the experiments. YZ performed the experiments, analyzed the data, made figures for the manuscript, wrote the manuscript, with contributions from JL and MZ. FT helped analyze the next generation sequencing and microbial data. YL was the pathologist who provided and evaluated the samples for identification of ECC.

### Conflict of interest statement

The authors declare that the research was conducted in the absence of any commercial or financial relationships that could be construed as a potential conflict of interest.
