# Supplementary material for: Comparative Analysis of the Microbial Profiles in Supragingival Plaque Samples Obtained From Twins With Discordant Caries Phenotypes and Their Mothers
Source: Front Cell Infect Microbiol. 2018 Oct 16;8:361. doi: 10.3389/fcimb.2018.00361 (PMC6232758; doi:10.3389/fcimb.2018.00361)
Supplement: Supplementary file 1 [file Data_Sheet_1.ZIP › Supplemental Materials/Supplemental Figure/Supplemental Figure.docx]

S 1 The alpha diversity was measured as Shannon index curve. Shannon index was used to evaluate the evenness of the microbial profiles.

S 2 Bacterial diversity in each sample was contributed by the following five phylum: Fusobacterium(44.8%), Bacteroidetes(21.7%), Actinobacteria(19.4%), Proteobacteria(4.9%) and Fimicutes(3.1%). Mother group showed high frequency of Bacteroidetes compared other two groups (P < 0.05).

S 3 Weighted UniFrac distances between monozygotic and dizygotic twins. The results confirmed that monozygotic twins shared a higher degree of similarity than dizygotic twins (P = 0.011).

S 4 Phylogenetic tree：species with similar color came from the same phylum. A total of 11 taxa at phylum level and 139 taxa at spices level were identified in all samples.

S 5 Metabolism activities in Caries and CariesFree group. The blue color represented Caries and orange was CariesFree. P-value was in an ascending order on the right side of the graph.

S 6 Metabolism activities in Caries and Mother group. The blue color represented Caries and green was Mother group. P-value was in an ascending order on the right side of the graph.

S 7 Metabolism activities in CariesFree and Mother group. The orange color represented CariesFree and green was Mother group. P-value was in an ascending order on the right side of the graph.
