# Supplementary material for: Comparative Analysis of the Microbial Profiles in Supragingival Plaque Samples Obtained From Twins With Discordant Caries Phenotypes and Their Mothers
Source: Front Cell Infect Microbiol. 2018 Oct 16;8:361. doi: 10.3389/fcimb.2018.00361 (PMC6232758; doi:10.3389/fcimb.2018.00361)
Supplement: Supplementary file 1 [file Data_Sheet_1.ZIP › Supplemental Materials/Supplemental Table.docx]

**Table 1. Summary of questionnaire.**

| **Twins NO.** | **Name** | **Sex** | **Age**  **(month)** | **Monozygotic**  **/Dizygotic twins** | **Daily Habbits** | **Oral Health Habbits** | **Eating**  **Habbits** |
| --- | --- | --- | --- | --- | --- | --- | --- |
| T.NO.1 | C.NO.1 | F | 57 | DZ | Same | Different | Same |
|  | C.F.NO.1 |  |  |  |  |  |  |
| T.NO.2 | C.NO.2 | F | 51 | DZ | Same | Same | Differnert |
|  | C.F.NO.2 | M |  |  |  |  |  |
| T.NO.3 | C.NO.3 | F | 71 | DZ | Same | Same | Different |
|  | C.F.NO.3 | M |  |  |  |  |  |
| T.NO.4  (triplet) | C.NO.4 | F | 66 | MZ | Same | Same | Same |
|  | C.F.NO.4 |  |  |  |  |  |  |
|  | C.NO.5 |  |  |  |  |  |  |
| T.NO.7 | C.NO.7 | F | 49 | DZ | Same | Same | Different |
|  | C.F.NO.7 |  |  |  |  |  |  |
| T.NO.8 | C.NO.8 | M | 70 | MZ | Same | Same | Same |
|  | C.F.NO.8 |  |  |  |  |  |  |
| T.NO.9 | C.NO.9 | M | 63 | DZ | Same | Same | Different |
|  | C.F.NO.9 |  |  |  |  |  |  |
| T.NO.10 | C.NO.10 | F | 56 | DZ | Same | Same | Same |
|  | C.F.NO.10 | M |  |  |  |  |  |
| T.NO.11 | C.NO.11 | F | 39 | DZ | Same | Same | Different |
|  | C.NO.11 |  |  |  |  |  |  |
| T.NO.12 | C.NO.12 | F | 53 | MZ | Same | Same | Same |
|  | C.F.NO.12 |  |  |  |  |  |  |
| T.NO.13 | C.NO.13 | F | 49 | MZ | Same | Same | Same |
|  | C.F.NO.13 |  |  |  |  |  |  |
| T.NO.14 | C.NO.14 | M | 64 | MZ | Same | Same | Same |
|  | C.F.NO14 |  |  |  |  |  |  |
| T.NO.15 | C.NO.15 | F | 54 | DZ | Same | Same | Different |
|  | C.F.NO.15 | M |  |  |  |  |  |
| T.NO.16 | C.NO.16 | M | 59 | MZ | Same | Same | Same |
|  | C.F.NO.16 |  |  |  |  |  |  |
| T.NO.17 | C.NO.17 | F | 51 | DZ | Same | Same | Different |
|  | C.F.NO17 | M |  |  |  |  |  |
