# Supplementary figures and images for: Comparative Analysis of the Microbial Profiles in Supragingival Plaque Samples Obtained From Twins With Discordant Caries Phenotypes and Their Mothers
Source: Front Cell Infect Microbiol. 2018 Oct 16;8:361. doi: 10.3389/fcimb.2018.00361 (PMC6232758; doi:10.3389/fcimb.2018.00361)

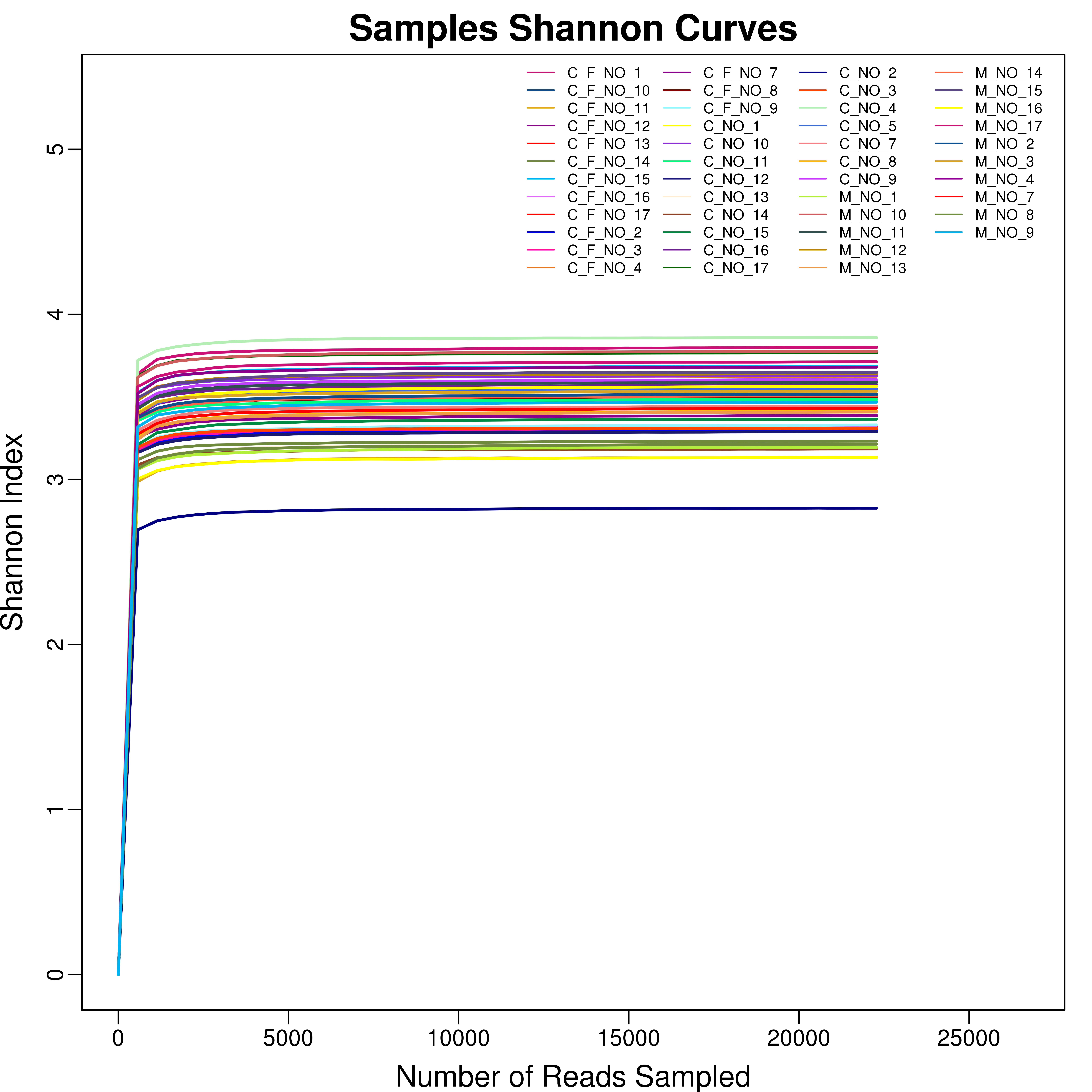

Supplement: Supplementary file 1 [file Data_Sheet_1.ZIP › Supplemental Materials/Supplemental Figure/S 1 Samples_Shannon_Curves.tif]

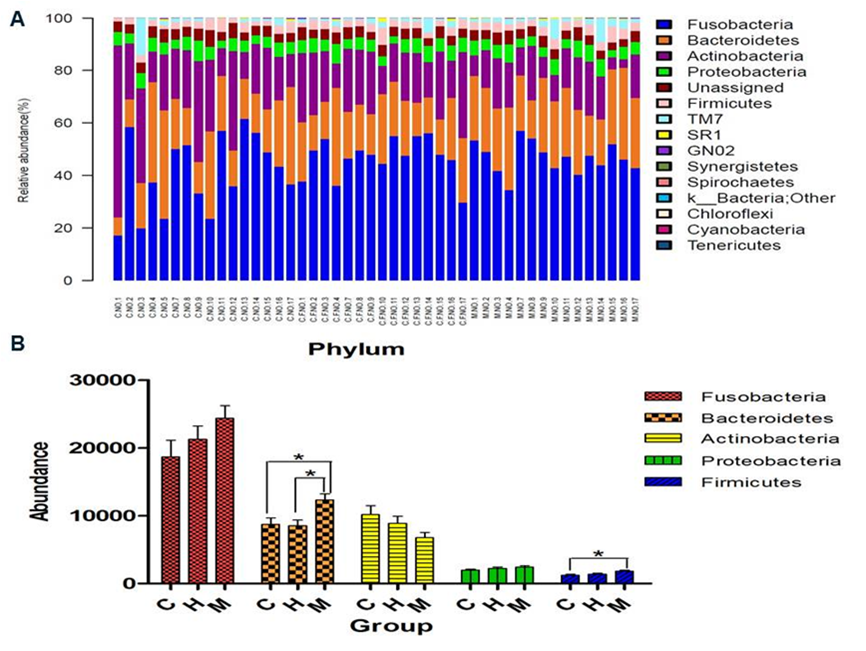

Supplement: Supplementary file 1 [file Data_Sheet_1.ZIP › Supplemental Materials/Supplemental Figure/S 2.png]

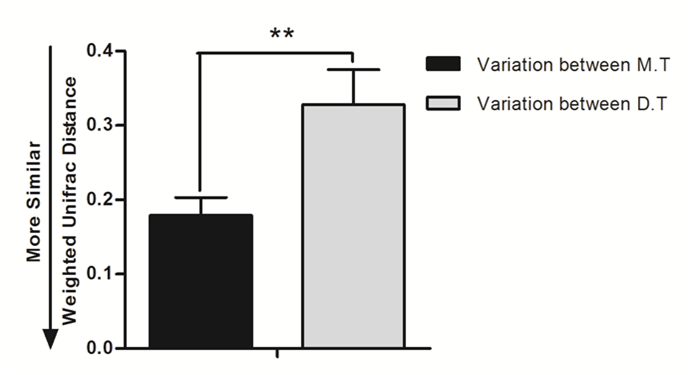

Supplement: Supplementary file 1 [file Data_Sheet_1.ZIP › Supplemental Materials/Supplemental Figure/S 3.png]

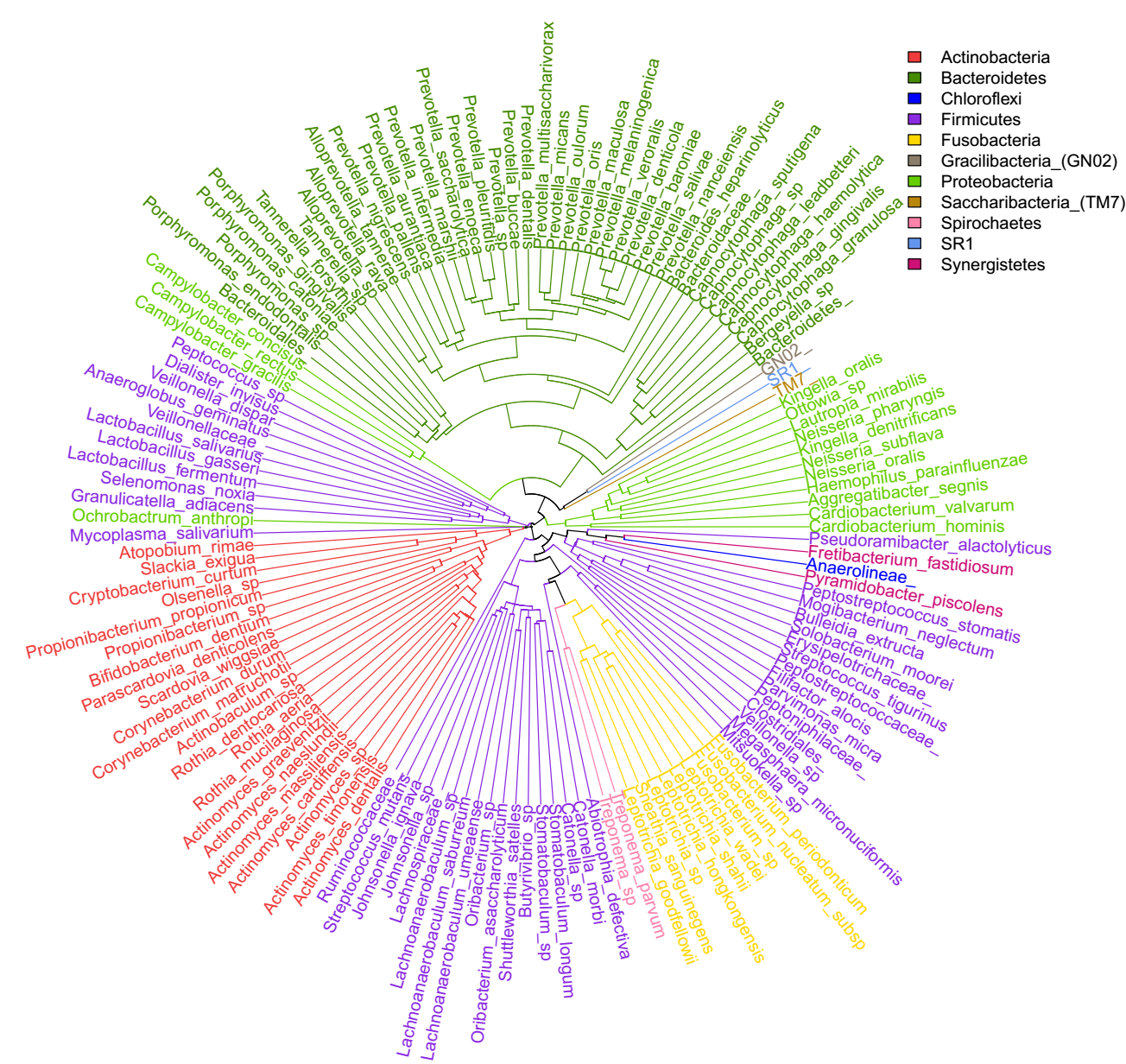

Supplement: Supplementary file 1 [file Data_Sheet_1.ZIP › Supplemental Materials/Supplemental Figure/S 4.png]

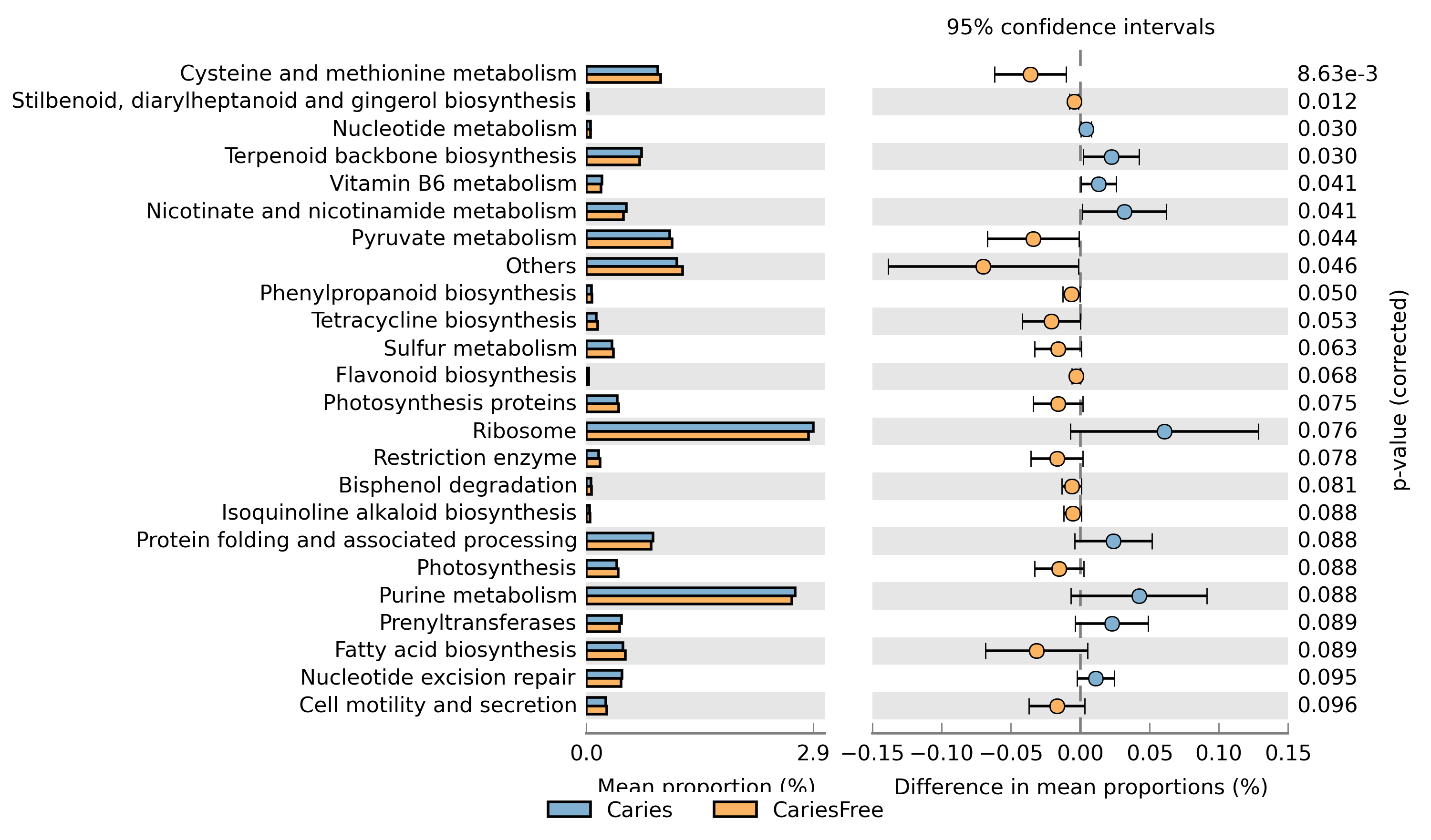

Supplement: Supplementary file 1 [file Data_Sheet_1.ZIP › Supplemental Materials/Supplemental Figure/S 5.png]

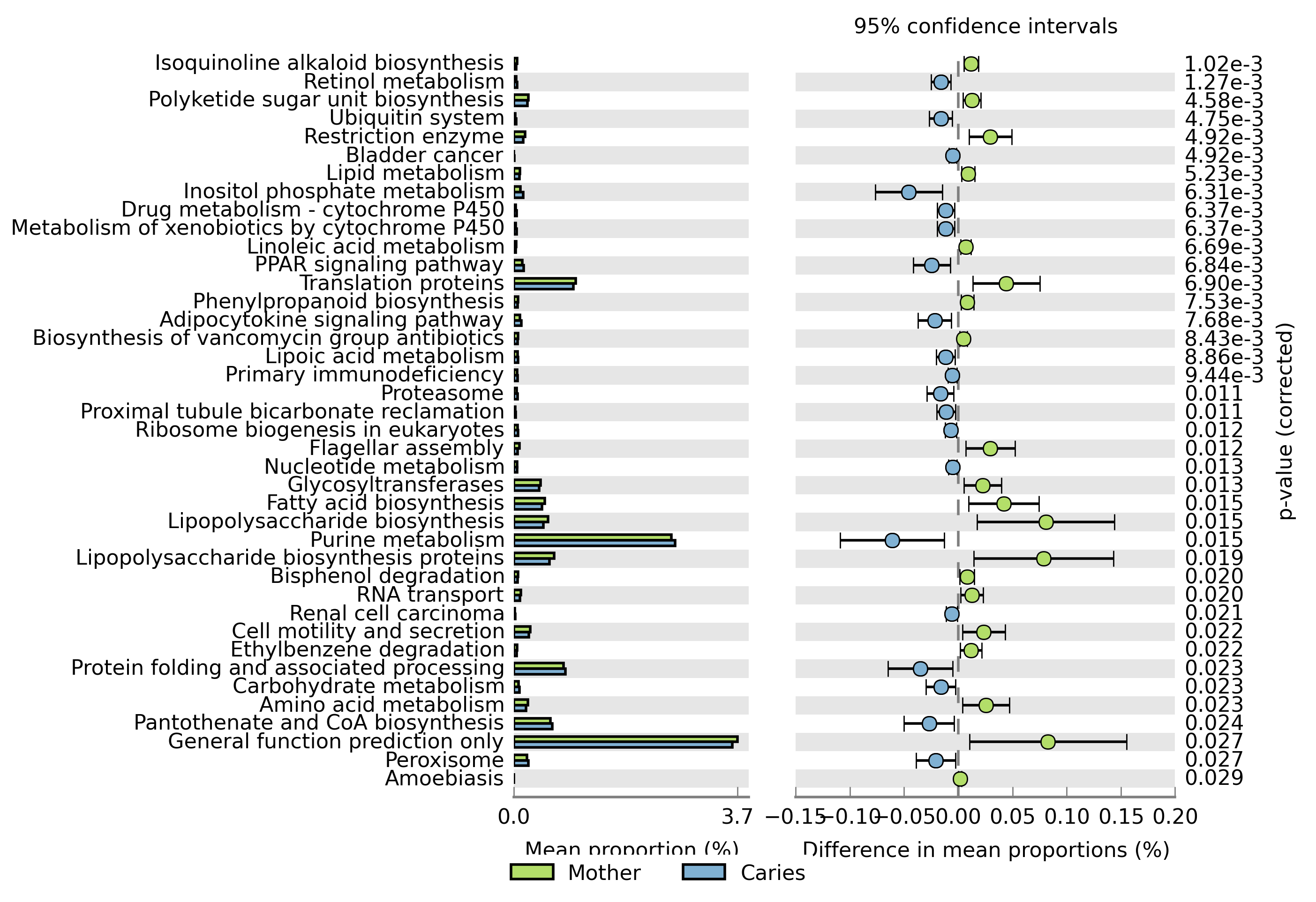

Supplement: Supplementary file 1 [file Data_Sheet_1.ZIP › Supplemental Materials/Supplemental Figure/S 6.png]

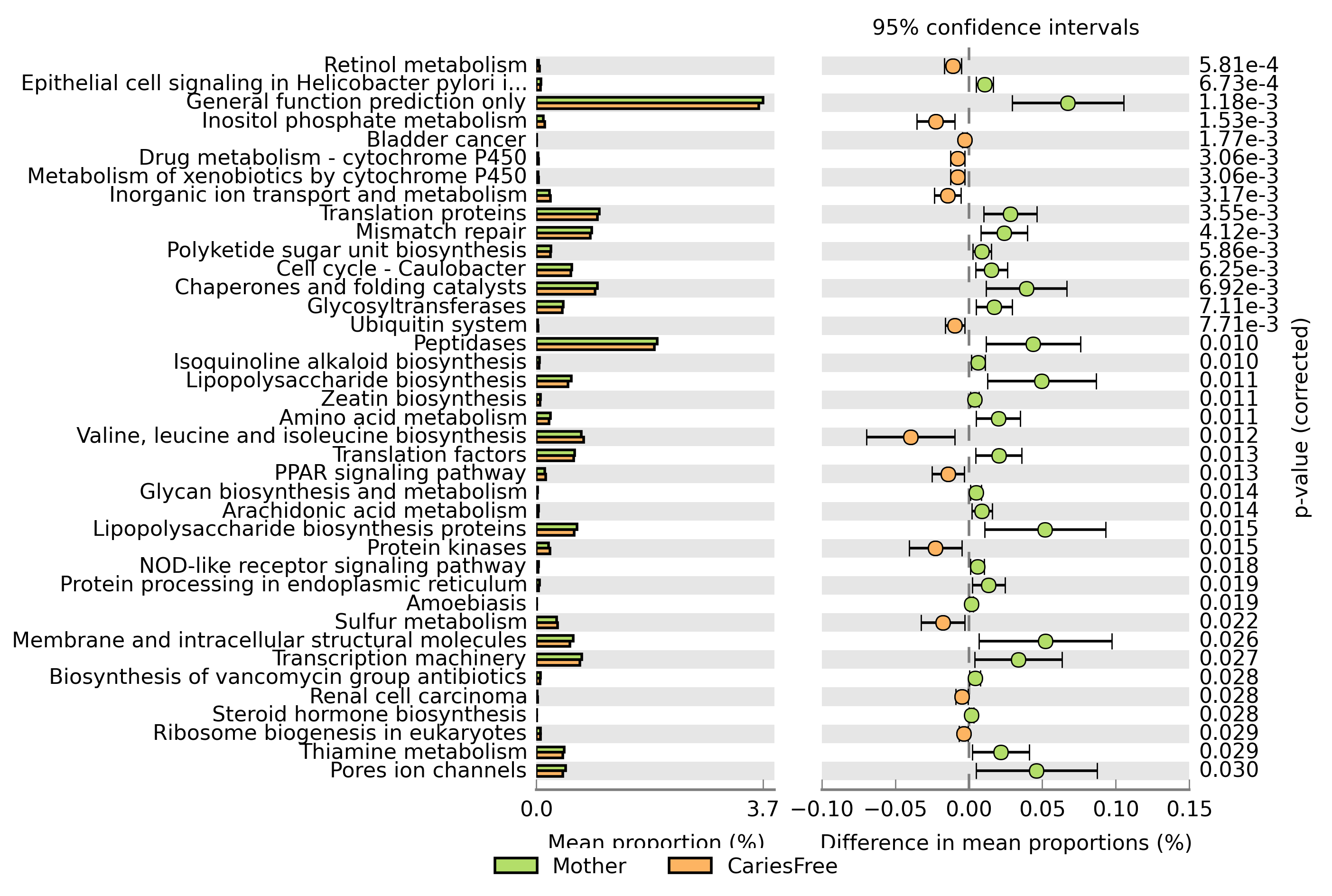

Supplement: Supplementary file 1 [file Data_Sheet_1.ZIP › Supplemental Materials/Supplemental Figure/S 7.png]
