# Supplementary material for: Comparative Analysis of the Microbial Profiles in Supragingival Plaque Samples Obtained From Twins With Discordant Caries Phenotypes and Their Mothers
Source: Front Cell Infect Microbiol. 2018 Oct 16;8:361. doi: 10.3389/fcimb.2018.00361 (PMC6232758; doi:10.3389/fcimb.2018.00361)
Supplement: Supplementary file 1 [file Data_Sheet_1.ZIP › Supplemental Materials/Supplemental Materials and Mothods.doc]

**Comparative analysis of the microbial profiles in supragingival plaque samples obtained from twins with discordant caries phenotypes and their mothers**

Yuqiao Zheng^1^, Meng Zhang^1^, Jin Li^1^, Han Jiang^1^, Fei Teng^1^, Yuhong Li^1^, Minquan Du^1*^

^1^ MOST KLOS & KLOBM, School & Hospital of Stomatology, Wuhan University, Luoyu Road 237, Wuhan City, Hubei, China.

* corresponding author

E-mail: [duminquan@whu.edu.cn](mailto:duminquan@whu.edu.cn)

**Total Bacterial Genomic DNA Extraction**

Bacteria in the plaque samples were collected by centrifugation at 13,200×g for 2 min. The bacteria were suspended in 480 µl of EDTA, 120 µl of lysozyme buffer and 2 µl of Mutanolysin and were then incubated at 37 °C for 60 min. The bacteria were centrifuged again and then suspended in lysis buffer and NaCl. Proteinase K was added, and the mixture was incubated for one hour at 55 °C and then inactivated at 95 °C. The samples were extracted with phenol-chloroform–isoamyl alcohol 3-4 times and centrifuged. DNA was isolated using Anhydrous ethanol and 70% cold ethanol. After the ethanol evaporated, the DNA was suspended in TE buffer and mixed with RNase for 1 h. DNA concentrations were measured using a NanoDrop Instrument (ThermoScientific, Willmington, DE, USA). All of the DNA samples were stored at −20 °C until further analysis.

**PCR and Pyrosequencing**

HOMINGS, which utilizes the speed and efficiency of next generation sequencing combined with the refinement of species-level identification. The procedures of HOMINGS in laboratory were carried out by a modified protocol. First, 10-50 ng of DNA through PCR amplification by using V3-V4 forward (341F) AATGATACGGCGACCACCGAGATCTACACTATGGTAATTGTCCTACGGGAGGCAGCAG and reverse (806R) CAAGCAAGAAGACGGCATACGAGA TNNNNNNNNNNNNAGTCAGTCAGCCGGACTACHVGGGTWTCTAAT primers.

Then, purified DNA library by using AMPure beads. Next, DNA library about 100 ng was pooled, gel-purified, and quantified using a bioanalyser and subsequently with qPCR. Finally, 12 PM of DNA library mixture was added 20％ Phix and then sequeced by use of Miseq ( Illumina, San Diego, CA).

**Sequencing Data Analysis**

**Schemetic representation of protocol for HOMINGS analyses**

An average of > 50,000 sequences of about 441 bp each sequence are typically obtained in a MiSeq run of about 90 samples. Bad reads are removed from analyses. Species - specific, 16S rRNA -based oligonucleotide "probes", many of which were originally designed for HOMIM, are used in a BLAST program (called ProbeSeq for HOMINGS) written by Sean Cotton to identify the frequency of oral bacterial targets. At present, 598 oligonucleotide probes of 17 to 40 bases target individual oral bacterial species or, in some cases, a few closely - related species. In order to get nearly complete coverage, an additional panel genus - specific probes is used. Raw sequence data such as FASTQ files can be download.

**Bioinformatic and statistical analysis**

Raw sequence data were quality-checked (denoised) with Ampliconnoise (Schloss et al. 2011) and chimeras removed with UCHIME(Edgar et al. 2011) using default settings. After quality checks, all analyses were performed using QIIME 1.9.0 software(Caporaso et al. 2010). High quality sequences were clustered into OTUs at 97% pairwise identity (OTU_0.03_) using the USEARCH(version 9.0) algorithm(Edgar 2013) with default settings, and representative sequences from each OTU_0.03_ were aligned against the Greengenes reference alignment(Desantis et al. 2006) using PyNAST(Caporaso, Bittinger, Bushman, Desantis, Andersen and Knight 2010). Aligned sequences were then used to build phylogenetic trees using the Fast Tree method(Price et al. 2009).

Taxonomy assignment of each representative sequencewas implemented using the BLAST algorithm (Altschul et al. 1997) against the Silva108 curated database (Pruesse et al. 2007). Sequences with reference sequence hit below 90% were called unclassified.

Alpha diversity of OTU libraries was described using the Chao1, Shannon, Simoson, Coverage, Observed species and ACE metrics as implemented in QIIME. Evenness was described by the Gini coefficient (Gcorr.), by calculating the area under the Lorenz curves per OTU_0.03_ pair (Wittebolle et al. 2009) with correction to minimize bias(Edwards et al. 2011).Alpha diversities between two communities were compared using a Student’s t-test in R software (R Development Core Team 2011). Distance matrices were constructed using the Unweighted and Weighted UniFrac algorithms in QIIME from the whole community phylogenetic tree. For statistical testing of sample groupings in a distance matrix, we applied ANOSIM with *P*-value less than 0.05 as significant. Significance of beta diversity for spatial heterogeneity of microbial communities was calculated with the algorithm described in elsewhere (Gülay and Smets 2015)in combination with Weighted UniFrac β diversity metric. Furthermore, in order to analysis the connection of microbiota and geographic characteristics,  β-diversity was analyzed using both Bray-Curtis and Jaccard in Vegan package (<https://cran.r-project.org/web/packages/vegan/index.html>) . The LEfSe analysis (http: //huttenhower.sph.havard.edu/galaxy) are used to find metagenomic biomaker and explanation in different groups. The figure illustrates in detail the for-mat of the input (a matrix with n rows and m columns) and the three steps performed by the computational tool: the KW rank sum test on classes, the pairwise Wilcoxon test between subclasses of different classes, and the LDA on the relevant features. PICRUSt is a bioinformatics software package designed to predict metagenome functional content from 16S rRNA survey (http://picrust.github.io/picrust). PICRUST (Huttenhower Lab, V 1.0.0) was used to recaptures key findings from KEGG and accurately predict the abundance of bacterial gene family in host associated communities. STAMP software package (v2.1.3) were used to for analyzing taxonomic or metabolic profiles that promotes ‘best practices’ in choosing appropriate statistical techniques and reporting results. The metabolic avtivities differences between different groups were evaluated using the Wilcoxon rank-sum analysis or Turkey-Kramer analysis of the variance (ANOVA) test, and with *P*-value less than 0.05 as significant.

**Statistical Analysis**

The demographic statistics (mean, median, standard error, range, and 95% confidence intervals) were generated for all samples. Paired-samples T test were used in comparing the bacterial abundance between caries and caries-free group at species level of HOMINGS general findings. Statistical significance tests were performed using the R program(v3.3.2). The differences in the diversity indexes or bacterial community patterns among caries, caries-free and mother groups were evaluated using the Wilcoxon rank-sum analysis or Kruskal-Wallis analysis of the variance (ANOVA) test, and were corrected by Benjamini-CHochberg. The software Metastats (http://metastats.cbcb.umd.edu/) are used to analysis the significant difference between groups of samples. The software R(v 3.3.2) is used to calibrate the p value, and the calibration method is 'Benjamini-CHochberg'. The prevalences of the taxa were compared using Fisher’s exact test. Principal coordinates analyses and the unweighted pair group method with arithmetic mean (UPGMA) were performed using the weighted Fast UniFrac web interface. Unless otherwise noted, significance was considered at an alpha value of 0.05.

**References**

Altschul SF, Madden TL, Schäffer AA, Zhang J, Zhang Z, Miller W, Lipman DJ. 1997. Gapped BLAST and PSI-BLAST: a new generation of protein database search programs. Nucleic Acids Res. 1997/09//;25:3389-3402.

Caporaso JG, Bittinger K, Bushman FD, Desantis TZ, Andersen GL, Knight R. 2010. PyNAST: a flexible tool for aligning sequences to a template alignment. Bioinformatics.26:266-267.

Desantis TZ, Hugenholtz P, Larsen N, Rojas M, Brodie EL, Keller K, Huber T, Dalevi D, Hu P, Andersen GL. 2006. Greengenes, a chimera-checked 16S rRNA gene database and workbench compatible with ARB. Applied & Environmental Microbiology.72:5069-5072.

Edgar RC. 2013. UPARSE: highly accurate OTU sequences from microbial amplicon reads. Nature Methods.10:996.

Edgar RC, Haas BJ, Clemente JC, Quince C, Knight R. 2011. UCHIME improves sensitivity and speed of chimera detection. Bioinformatics.27:2194.

Edwards A, Anesio AM, Rassner SM, Sattler B, Hubbard B, Perkins WT, Young M, Griffith GW. 2011. Possible interactions between bacterial diversity, microbial activity and supraglacial hydrology of cryoconite holes in Svalbard. Isme Journal.5:150-160.

Gülay A, Smets BF. 2015. An improved method to set significance thresholds for β diversity testing in microbial community comparisons. Environmental Microbiology.17:3154-3167.

Price MN, Dehal PS, Arkin AP. 2009. FastTree: Computing Large Minimum Evolution Trees with Profiles instead of a Distance Matrix. Molecular Biology and Evolution.26:1641-1650.

Pruesse E, Quast C, Knittel K, Fuchs BM, Ludwig W, Peplies J, Glöckner FO. 2007. SILVA: a comprehensive online resource for quality checked and aligned ribosomal RNA sequence data compatible with ARB. Nucleic Acids Res.35:7188-7196.

Schloss PD, Gevers D, Westcott SL. 2011. Reducing the Effects of PCR Amplification and Sequencing Artifacts on 16S rRNA-Based Studies. Plos One.6:e27310.

Wittebolle L, Marzorati M, Clement L, Balloi A, Daffonchio D, Heylen K, Vos PD, Verstraete W, Boon N. 2009. Initial community evenness favours functionality under selective stress. Nature.458:623-626.
